# Lipid Droplets in Poultry Lipid Metabolism: Characteristics, Regulatory Factors, and Implications for Fatty Liver and Muscle Lipid Deposition

**DOI:** 10.3390/ani16192992

**Published:** 2026-09-23

**Authors:** Yiqin Yan, Lupeng Chen

**Affiliations:** College of Life Science and Resources and Environment, Yichun University, Yichun 336000, China

**Keywords:** lipid droplets, poultry, fatty liver, organelle interactions, nutritional regulation

## Abstract

Lipid droplets (LDs) are dynamic metabolic platforms that integrate neutral-lipid storage, fatty-acid trafficking, organelle communication, cellular energy metabolism, and stress adaptation in poultry cells. Lipid metabolism plays a central role in poultry growth, reproductive function, production performance, meat quality, and metabolic health. Unlike mammals, de novo lipogenesis in poultry occurs predominantly in the liver, making hepatic lipid homeostasis particularly important for maintaining physiological function and production efficiency. Dysregulation of lipid metabolism contributes to excessive fat deposition, fatty liver hemorrhagic syndrome (FLHS), oxidative stress, and impaired meat quality in poultry production systems. At the cellular level, LDs partition fatty acids among neutral-lipid storage, VLDL assembly, membrane synthesis, mitochondrial oxidation, and stress-buffering pathways. Poultry studies show that hepatic LD accumulation and altered lipid handling are closely associated with fatty liver and muscle lipid deposition, while mechanistic studies from mammalian and cellular models provide useful hypotheses for LD turnover and LD–organelle communication. Because direct poultry-specific evidence for several LD–organelle mechanisms remains limited, these mechanisms are discussed cautiously as conceptual frameworks and future research directions rather than as established causal pathways in poultry. Nutritional interventions, including balanced dietary energy, fatty acid sources, amino acid balance, choline, methionine, antioxidants, trace elements, phytochemicals, probiotics, and bile acids, can influence LD-associated lipid handling and improve metabolic health in poultry. In this review, we summarize current evidence on LD formation, turnover, and LD-associated regulatory pathways in poultry lipid metabolism, with particular emphasis on fatty liver, muscle lipid deposition, and nutritional regulation. We further discuss unresolved questions and future perspectives for defining the causal roles of LD dynamics in poultry health and production. The review therefore treats laying hens and broilers as distinct physiological contexts: estrogen-driven VLDLy production and susceptibility to fatty liver hemorrhagic syndrome in laying hens, versus hepatic lipid export, abdominal fat deposition, muscle lipid utilization, and meat-quality formation in broilers.

## Simple Summary

Poultry produce much of their body fat in the liver. Lipid droplets are small structures inside cells that temporarily store fat and help determine whether it is used for energy, transported to the ovary or muscle, or retained in the liver. When this balance is disturbed, laying hens may develop fatty liver hemorrhagic syndrome, while broilers may show excessive abdominal fat or reduced meat quality. This review summarizes how lipid droplets are formed, broken down, and connected with other cell structures, and how diet may influence these processes. Current poultry evidence is still limited, especially for detailed cellular mechanisms. Further research on lipid droplet structure, turnover, and function may help improve poultry health, reproduction, and product quality.

## 1. Introduction

Lipid metabolism is a critical physiological process regulating energy storage, membrane biosynthesis, reproduction, stress adaptation, and production performance in poultry [1,2,3]. Excessive or abnormal lipid accumulation is closely associated with metabolic disorders such as fatty liver hemorrhagic syndrome (FLHS), obesity, oxidative stress, and reduced reproductive performance in laying hens, as well as undesirable carcass fat deposition and impaired meat quality in broilers [4]. In recent decades, intensive genetic selection for rapid growth or high egg production has increased metabolic demand, although modern broiler selection has also improved feed efficiency and reduced carcass adiposity [5].

Unlike mammals, in which adipose tissue is a major site of de novo lipogenesis, lipid synthesis in poultry occurs predominantly in the liver [1]. Hepatic lipids are subsequently exported as very low-density lipoproteins (VLDLs) and transported to peripheral tissues, including adipose tissue, skeletal muscle, and ovaries [3]. This unique metabolic characteristic makes the poultry liver particularly susceptible to excessive lipid accumulation and oxidative damage under conditions of nutritional imbalance, environmental stress, or metabolic overload [6]. Furthermore, intramuscular fat (IMF) deposition is an important determinant of poultry meat quality, influencing tenderness, juiciness, and flavor [7]. Therefore, understanding the regulatory mechanisms governing lipid metabolism is of considerable importance for improving poultry production efficiency and product quality.

The biological purpose of hepatic lipid trafficking differs fundamentally between poultry production types. In laying hens, estrogen drives intense hepatic synthesis of triacylglycerol, vitellogenin, and yolk-targeted VLDLy to sustain follicular growth and egg formation; this physiological hyperlipogenic state also creates susceptibility to hepatic lipid retention and FLHS when lipid synthesis exceeds export and stress-buffering capacity [1,3,4]. In broilers, hepatic lipid output is directed mainly toward rapid somatic growth, abdominal adipose storage, and skeletal-muscle lipid supply, making carcass fatness and meat quality the principal production concerns [5,8]. Waterfowl are considered separately as comparative models of unusually high hepatic lipid-storage tolerance [9,10].

Lipid droplets (LDs) are phospholipid-monolayer organelles whose neutral-lipid core and protein-coated surface form a dynamic metabolic platform. By recruiting lipid-synthetic enzymes, lipases, trafficking factors, and stress-response proteins, LDs integrate lipid storage, mobilization, cellular energy metabolism, and stress adaptation [11,12,13,14,15]. LDs can interact with other organelles, including the endoplasmic reticulum (ER), mitochondria, peroxisomes, and lysosomes, thereby influencing lipid homeostasis and metabolic adaptation [16,17,18].

Mechanistic studies in mammalian and cellular models indicate that dysregulated LD dynamics can contribute to hepatic steatosis, mitochondrial dysfunction, oxidative stress, inflammation, and abnormal lipid deposition [19,20]. Poultry studies more often provide association-level evidence linking hepatic steatosis, oxidative stress, and altered lipid handling, and direct LD-specific causality remains limited [21,22,23]. In skeletal muscle, LDs are closely associated with mitochondrial oxidative capacity and fatty acid utilization, providing a mechanistic hypothesis for energy metabolism and meat-quality formation [24]. Nutritional factors such as dietary fatty acids, methyl donors, antioxidants, and phytochemicals may influence LD-associated lipid handling and metabolic health in poultry, although most studies have not directly measured LD dynamics [25].

Although increasing attention has been paid to poultry lipid metabolism, most studies have focused primarily on lipid synthesis and fat deposition, whereas the biological functions and regulatory mechanisms of LDs remain poorly understood in poultry species. In particular, direct evidence for LD–organelle interactions in avian tissues is still limited, and LD-based mechanisms established in mammals or cell models should not be directly extrapolated to poultry without validation.

In this review, we summarize current evidence on LD biology and dynamics in poultry, with emphasis on hepatic steatosis, muscle lipid deposition, and nutritional regulation. We also discuss LD–organelle interactions as a potential mechanistic framework for future poultry research, while distinguishing poultry-derived evidence from mechanisms inferred from mammalian and cellular models.

It should be noted that poultry-specific studies directly examining LD proteins, LD turnover, and LD–organelle contact sites are still limited. Therefore, this review distinguishes three levels of evidence: first, poultry studies showing lipid accumulation, hepatic steatosis, IMF deposition, or nutritional regulation; second, poultry studies implicating LD-associated genes or lipid metabolic pathways; and third, mechanistic studies from mammalian or cellular models that may provide hypotheses for poultry research. This distinction is important to avoid overinterpreting LD accumulation as direct evidence of LD-mediated causality. The liver-centered partitioning of hepatic TAG among LD storage, VLDL/VLDLy export, and fatty-acid oxidation, together with the evidence hierarchy used in this review, is summarized in Figure 1.

## 2. LD Biology and Dynamics in Poultry

### 2.1. Formation and Growth of LDs

LDs are evolutionarily conserved organelles that store neutral lipids, mainly triacylglycerols (TAGs) and cholesteryl esters, within a hydrophobic core surrounded by a phospholipid monolayer. Unlike bilayer-bound organelles, LDs originate from the endoplasmic reticulum (ER), where neutral lipids accumulate between the two leaflets of the ER membrane and subsequently bud toward the cytoplasm [13,26,27,28]. This unique structure allows LDs to function not only as lipid reservoirs but also as dynamic platforms involved in lipid synthesis, storage, mobilization, and cellular stress responses [11,18,29].

The formation of LDs is closely associated with TAG synthesis [30,31]. In most cells, fatty acids are first activated to acyl-CoA by acyl-CoA synthetases and then incorporated into glycerolipids through sequential enzymatic reactions [30]. Diacylglycerol acyltransferase 1 and 2 (DGAT1 and DGAT2) catalyze the final and rate-limiting step of TAG synthesis, thereby directly contributing to LD biogenesis and expansion [14]. DGAT2 is frequently associated with the ER and LD surface, suggesting that TAG synthesis and LD growth are spatially coordinated [32,33]. In addition, several structural proteins, including seipin [34,35,36,37], fat storage-inducing transmembrane proteins (FIT proteins) [38], and cell death-inducing DFFA-like effector (CIDE) family proteins [39,40], participate in LD nucleation, budding, and growth.

In poultry, hepatic lipid synthesis is particularly important because the liver is the primary site of de novo lipogenesis. The newly synthesized fatty acids are esterified into TAGs, incorporated into LDs, or packaged into very low-density lipoproteins (VLDLs) for transport to peripheral tissues [1]. Therefore, LD formation in avian hepatocytes is tightly linked to whole-body lipid distribution, egg yolk precursor synthesis, abdominal fat deposition, and the development of fatty liver. Excessive TAG synthesis or impaired lipid export may lead to abnormal LD accumulation in hepatocytes, which is a characteristic feature of hepatic steatosis and fatty liver hemorrhagic syndrome in laying hens.

LD growth is regulated not only by TAG availability but also by LD-associated proteins [41]. The perilipin family proteins are among the best-characterized LD coat proteins and play important roles in controlling lipid storage and mobilization [42]. PLIN2 is widely expressed in non-adipose tissues and is commonly associated with intracellular lipid accumulation, whereas PLIN5 is enriched in oxidative tissues and has been linked to LD–mitochondria coupling and fatty acid oxidation [43]. Although the functions of perilipins have been extensively investigated in mammals, their specific roles in poultry liver, muscle, and reproductive tissues remain insufficiently characterized. Understanding how LD-associated proteins regulate lipid storage and mobilization in poultry may provide new insights into the control of fatty liver and meat quality.

PLIN2 and PLIN3 function as LD-surface gatekeepers rather than passive coat proteins. PLIN2 preferentially associates with mature LDs, stabilizes the phospholipid monolayer, and can restrict access of cytosolic lipases and autophagic machinery, thereby favoring neutral-lipid retention. PLIN3 is recruited rapidly to nascent and small LDs and to diacylglycerol-enriched ER membranes, where it contributes to early LD coating, protein targeting, and maturation [44,45]. PLIN5 is more prominent in oxidative tissues and links LD storage to regulated fatty-acid use. Poultry studies have detected perilipin-family expression in liver, muscle, and adipose tissues, but the tissue-specific causal functions of PLIN2 and PLIN3 in avian LD turnover remain largely unresolved.

DGAT1 and DGAT2 catalyze the same terminal reaction in TAG synthesis but occupy partially distinct metabolic positions. In mammalian hepatocytes, DGAT2 preferentially esterifies nascent diacylglycerol and de novo synthesized fatty acids on the cytosolic face of the ER, supporting local TAG synthesis and LD expansion, whereas DGAT1 can re-esterify preformed fatty acids and contributes to complete lipidation and maturation of ApoB-containing VLDL particles within the ER [46,47]. These functions overlap, and DGAT2 can still support ApoB particle secretion when DGAT1 is absent. Consequently, a simple assignment of DGAT1 to export and DGAT2 to storage would be premature in poultry; isoform-specific tracing and loss-of-function studies in avian hepatocytes are required.

The principal structural and regulatory proteins that may control LD formation, stability, mobilization, and metabolic coupling in poultry liver and skeletal muscle are summarized in Table 1.

### 2.2. LD Degradation: Lipolysis and Lipophagy

LDs are not static lipid stores. Under energy-demanding or stress conditions, stored TAGs can be hydrolyzed to release fatty acids for mitochondrial or peroxisomal oxidation [59]. Classical cytosolic lipolysis is mainly mediated by adipose triglyceride lipase (ATGL), hormone-sensitive lipase (HSL), and monoacylglycerol lipase (MGL), which sequentially hydrolyze TAGs to diacylglycerols, monoacylglycerols, and free fatty acids [55]. Among these enzymes, ATGL catalyzes the first and rate-limiting step of TAG hydrolysis and is therefore essential for initiating LD breakdown [55].

In addition to classical lipolysis, LDs can also be degraded through autophagy-dependent pathways, collectively termed lipophagy [56]. During lipophagy, LDs are recognized by autophagic machinery and delivered to lysosomes, where neutral lipids are hydrolyzed by lysosomal acid lipase [56]. Lipophagy is particularly important in hepatocytes, where it contributes to lipid clearance and protects against excessive steatosis. Studies in hepatocyte models have shown that LD catabolism can involve both cytosolic lipolysis and selective autophagic degradation, indicating that these two pathways may cooperate to maintain lipid homeostasis [60].

For poultry, LD degradation has important physiological and production-related implications, but the available evidence remains limited. In mammalian hepatocytes, lipophagy contributes to hepatic LD clearance and protects against excessive steatosis [61,62]. Whether insufficient LD degradation directly aggravates fatty liver in laying hens remains insufficiently investigated. Nevertheless, given the high hepatic lipogenic burden in laying hens, LD turnover may represent a relevant but underexplored mechanism in FLHS. In broilers, studies have mainly addressed abdominal fat deposition, feed efficiency, and meat lipid metabolism rather than LD turnover itself [8,63]. Therefore, direct evidence for ATGL-mediated lipolysis or lipophagy in broiler tissues remains limited. Future studies should determine whether nutritional interventions, genetic background, or environmental stress regulate poultry lipid metabolism through LD degradation pathways.

### 2.3. Characteristics of Lipid Metabolism in Poultry

A key feature of poultry lipid metabolism is that the liver, rather than adipose tissue, is the major site of de novo fatty acid synthesis. Hepatic lipogenesis in chickens includes fatty acid synthesis, TAG synthesis, and export of synthesized lipids to peripheral tissues [8]. This metabolic pattern makes avian species particularly dependent on hepatic lipid homeostasis. When hepatic lipid synthesis exceeds the capacity for lipid oxidation or VLDL secretion, TAGs accumulate in hepatocytes as LDs, predisposing birds to fatty liver [64].

In laying hens, hepatic lipid metabolism is further intensified by egg production. Estrogen stimulates the synthesis of yolk-targeted VLDL and vitellogenin, which are transported from the liver to the ovary for follicular development and yolk deposition [3]. This physiological adaptation is essential for reproduction but also increases the metabolic burden on the liver. Under high-energy diets or inappropriate energy-to-protein balance, hepatic lipid export and metabolic capacity may become insufficient, leading to excessive LD accumulation and liver injury [64,65]. Environmental stress, including heat stress, may further disturb oxidative balance and lipid metabolism, but its direct relationship with hepatic LD dynamics in laying hens requires further investigation.

The transition from sexual immaturity to the onset of lay represents a particularly important stage of hepatic lipid remodeling in female poultry. Rising estrogen concentrations reprogram the liver toward the synthesis of vitellogenin, apolipoprotein VLDL-II, yolk-targeted VLDLy particles, and large quantities of yolk lipids [3]. During this transition, hepatic LDs may act as temporary buffering compartments that accommodate the rapid increase in TAG synthesis and partition newly synthesized lipids between intracellular storage and VLDLy assembly. Proper coordination among LD biogenesis, LD turnover, phospholipid availability, and lipoprotein export may therefore contribute to timely yolk deposition and reproductive maturation. Conversely, insufficient body lipid reserves, inadequate hepatic lipid synthesis, or defective partitioning between LD storage and VLDLy secretion could impair prelay metabolic adaptation. Direct longitudinal evidence describing hepatic LD number, size, composition, and associated proteins during the transition from the pullet stage to the onset of lay remains scarce, making this an important direction for future research.

Broilers exhibit another lipid metabolic pattern. Modern genetic selection has markedly improved growth rate, breast muscle yield, feed efficiency, and carcass leanness, while selection against excessive abdominal fat has reduced overall adiposity [5,66]. Excessive fat deposition should therefore not be considered a universal consequence of contemporary broiler genetics. An associated challenge may instead arise in broiler breeder parents, in which limited body-fat accumulation during rearing can compromise the metabolic preparation for sexual maturation, the onset of lay, and subsequent reproductive performance. In commercial broilers, hepatic lipogenesis remains an important contributor to lipid partitioning among abdominal adipose tissue and skeletal muscle, and excessive energy intake can still promote abdominal fat deposition [8,63,66]. Because abdominal fat has low economic value and is often undesirable, regulation of hepatic lipid synthesis and LD dynamics remains relevant to carcass quality, whereas breeder hens require sufficient lipid reserves to support reproductive maturation [8,66].

Waterfowl, especially ducks and geese, show a remarkable capacity for hepatic lipid deposition. This characteristic has been used in fatty liver production models, but it also provides a useful system for studying hepatic LD expansion, lipid storage tolerance, and metabolic adaptation [9,10]. Compared with chickens, waterfowl may exhibit distinct mechanisms of hepatic lipid handling, including enhanced TAG storage, altered lipid export, and specific responses to overfeeding [9,10,67]. Understanding LD biology in different poultry species may therefore help explain species-specific differences in lipid accumulation and metabolic resilience.

Overall, the unique lipid metabolic features of poultry suggest that LDs provide a useful cellular framework for understanding how lipid storage, mobilization, oxidation, and export are coordinated in avian tissues. In the liver, LD-associated lipid handling may influence whether excess fatty acids are stored, exported, oxidized, or converted into lipotoxic intermediates [11,14]. In skeletal muscle, LDs may contribute to fatty acid utilization and meat quality-related lipid deposition [24]. In adipose tissue, LDs are closely related to fat deposition and energy storage [8,66]. Therefore, LD biology provides a useful but still developing framework for understanding poultry fatty liver, fat deposition, and nutritional regulation.

## 3. Roles of LDs in Poultry Fatty Liver and Muscle Lipid Deposition

### 3.1. LDs in Poultry Fatty Liver

Fatty liver hemorrhagic syndrome (FLHS) is one of the most important metabolic disorders in laying hens, particularly during the peak laying period. It is characterized by excessive hepatic lipid accumulation, liver enlargement, reduced egg production, hepatic hemorrhage, and sudden death [6,68]. Because the liver is the major site of de novo lipogenesis in poultry, hepatocytes are continuously exposed to a high lipid synthetic load [1,69]. Under physiological conditions, newly synthesized fatty acids are esterified into triacylglycerols (TAGs), transiently stored in LDs, or exported as very low-density lipoproteins (VLDLs) [1,66,70]. When lipid synthesis exceeds the capacity of lipid oxidation and lipoprotein secretion, TAGs accumulate within hepatocytes in the form of enlarged LDs, which is a hallmark of hepatic steatosis and FLHS [4,21]. Recent studies further support that FLHS involves not only lipid overaccumulation but also insulin resistance, metabolic blockade, oxidative stress, inflammatory responses, mitochondrial dysfunction, epigenetic remodeling, and gut–liver axis disturbance [22,23].

Importantly, hepatic LD accumulation is a histological and biochemical feature of poultry fatty liver, but LD accumulation alone does not prove that LDs causally regulate disease progression. Mechanistic relevance should be inferred only when LD biogenesis, LD turnover, LD-associated proteins, VLDL export, lipophagy, or LD–mitochondria coupling are shown to alter lipid flux, oxidative stress, or hepatocellular injury. Therefore, this section distinguishes LD accumulation as a feature of fatty liver from LD-associated processes that may contribute to disease development.

#### 3.1.1. Estrogen-Driven Physiological Hyperlipogenesis and Pathological Lipid Retention

Estrogen-induced hepatic lipogenesis is not inherently pathological in laying hens. During sexual maturation and active laying, estrogen increases fatty-acid and TAG synthesis while simultaneously stimulating ApoB, microsomal triglyceride-transfer protein (MTP), vitellogenin, and VLDLy production for ovarian uptake [3]. The system remains physiological when newly synthesized TAG is efficiently incorporated into VLDLy and exported to developing follicles [3]. A pathological shift may occur when hepatic synthesis exceeds VLDLy assembly, phosphatidylcholine availability, antioxidant capacity, or mitochondrial and peroxisomal oxidative utilization [4,64,71]. Under these conditions, TAG is increasingly retained in enlarged LDs, and the same endocrine program that sustains egg production becomes a metabolic vulnerability [4,64].

From a cellular perspective, LD accumulation in hepatocytes may initially represent an adaptive response to lipid overload. By esterifying excess fatty acids into TAGs and storing them in LDs, cells can reduce exposure to lipotoxic intermediates such as free fatty acids, diacylglycerols, and ceramides [20]. This protective role of TAG storage has been demonstrated in mammalian cells, where LD formation can buffer fatty acid-induced lipotoxicity [20]. However, persistent or excessive LD accumulation may become pathological when LD expansion is accompanied by impaired lipolysis, defective lipophagy, reduced mitochondrial β-oxidation, endoplasmic reticulum stress, oxidative damage, and inflammatory activation [19,72]. Therefore, hepatic LDs should not be regarded simply as passive markers of steatosis. Instead, LD-associated processes may influence whether lipid overload is safely stored, metabolically utilized, or linked to cellular injury, although direct causal evidence in poultry remains incomplete [19].

#### 3.1.2. From Adaptive Steatosis to Hemorrhagic Injury

The transition from steatosis to hepatic hemorrhage is best viewed as a proposed multi-hit process rather than a direct consequence of LD abundance. Persistent lipid loading causes hepatocyte swelling, liver enlargement, and tissue friability, while mitochondrial ROS, phospholipid and PUFA peroxidation, inflammatory cytokines, and sinusoidal microcirculatory disturbance weaken parenchymal and vascular integrity [4,6,21,22,23,68,73]. Extracellular-matrix remodeling and reduced mechanical resilience may further sensitize the enlarged liver to rupture during egg laying, handling, or increased abdominal pressure [6,68]. However, the complete molecular sequence linking LD expansion to vascular failure and hemorrhage has not yet been demonstrated experimentally in poultry, and individual steps should therefore be tested rather than assumed.

Several molecular pathways may contribute to abnormal LD accumulation in poultry fatty liver. First, enhanced hepatic lipogenesis increases the supply of fatty acids and TAGs for LD formation. Lipogenic transcription factors and enzymes, including sterol regulatory element-binding protein 1c (SREBP1c), acetyl-CoA carboxylase (ACC), fatty acid synthase (FASN), stearoyl-CoA desaturase (SCD), and DGATs, are generally associated with increased hepatic lipid synthesis and TAG storage [22]. Second, impaired VLDL assembly or secretion may limit lipid export from the liver, thereby increasing intracellular LD retention [1,3,4]. This mechanism is especially relevant in laying hens, in which estrogen-driven yolk precursor synthesis requires intensive hepatic lipid export to the ovary [3]. Third, insufficient LD degradation may promote hepatic steatosis if reduced cytosolic lipolysis or impaired lipophagy prevents LD turnover and limits fatty acid mobilization for oxidation or secretion; however, this possibility still requires direct validation in poultry [72].

#### 3.1.3. ER Stress as a Metabolic Tipping Point

Because LDs originate from the ER and VLDL is assembled within the ER lumen, ER homeostasis is a critical determinant of whether hepatic TAG is stored or exported [13,74]. Sustained lipid synthesis can activate the PERK–eIF2α–ATF4–CHOP, IRE1–XBP1, and ATF6 branches of the unfolded-protein response [74,75]. Initially, these pathways may expand folding and lipid-synthetic capacity, but chronic ER stress can impair ApoB folding, MTP-dependent lipidation, phosphatidylcholine supply, and VLDL secretion while enhancing inflammatory and apoptotic signaling [74,75]. Reduced export then redirects additional TAG toward cytosolic LDs, generating a feed-forward cycle of ER stress, LD expansion, and hepatocellular injury [74,75]. Direct measurements of unfolded-protein-response activity together with VLDLy secretion are needed to establish this tipping point in laying hens.

LD-associated proteins are important regulators of hepatic LD stability and metabolism. Perilipin 2 (PLIN2) is widely expressed in non-adipose tissues and is commonly associated with LD accumulation [43,48]. Increased PLIN2 expression may stabilize LDs and protect them from lipase-mediated degradation, thereby favoring lipid storage [43,48,76]. In contrast, PLIN5 is enriched in oxidative tissues and has been linked mainly in mammalian studies to coordination between LD storage and mitochondrial fatty acid oxidation [61,72]. Although the specific roles of PLIN proteins in poultry fatty liver remain incompletely defined, their known functions in LD stabilization, lipase access, and organelle coupling suggest that they are candidate regulators of avian hepatic lipid homeostasis [43]. Similarly, DGAT1/2 promote TAG synthesis and LD growth, whereas ATGL and HSL mediate LD breakdown [55]. An imbalance between lipid storage and lipid mobilization pathways may contribute to LD expansion in FLHS, but the causal contribution of each LD-associated protein needs poultry-specific functional validation [77].

Oxidative stress and mitochondrial dysfunction are closely linked to LD dysregulation in poultry fatty liver. In FLHS models induced by high-energy or nutritionally imbalanced diets, hepatic lipid accumulation is frequently accompanied by altered glucose, lipid, and amino acid metabolism, reduced antioxidant capacity, and inflammatory activation [22,78]. Excessive LD accumulation may increase the demand for mitochondrial fatty acid oxidation, resulting in electron transport chain overload and reactive oxygen species production. Conversely, mitochondrial dysfunction reduces fatty acid oxidation capacity, further aggravating hepatic LD accumulation. This creates a vicious cycle among LD expansion, mitochondrial impairment, oxidative stress, and hepatocellular injury [72]. Taurine supplementation has been reported to protect against FLHS in laying hens partly through regulation of mitochondrial homeostasis, including mitochondrial dynamics, autophagy and antioxidant capacity, supporting the importance of mitochondria-related mechanisms in poultry fatty liver [73].

#### 3.1.4. Programmed Cell Death in Avian Hepatocytes

Programmed cell death may connect lipotoxic stress to irreversible liver injury. Among the candidate pathways, mitochondrial apoptosis currently has the strongest avian support: HELP-diet and intervention studies in laying hens have linked mitochondrial damage and defective autophagy with hepatocyte apoptosis, and dioscin-mediated activation of ERα-AMPK-dependent mitophagy reduced apoptosis and FLHS severity [79]. Pyroptosis is mechanistically plausible because lipid overload and damaged mitochondria can activate the NLRP3-caspase-1-GSDMD axis, whereas ferroptosis is plausible because hepatic PUFA enrichment, iron-dependent lipid peroxidation, GPX4 depletion, and impaired SLC7A11 defense can cause membrane failure [80,81]. Nevertheless, direct evidence that pyroptosis or ferroptosis drives FLHS is currently insufficient. Future poultry studies should combine morphology and functional rescue with cleaved caspase-3, NLRP3, cleaved caspase-1, GSDMD-N, ACSL4, GPX4, SLC7A11, labile iron, and lipid-peroxidation measurements.

#### 3.1.5. Lipophagy as a Hepatic Defense Mechanism

Lipophagy provides a second route, alongside ATGL-mediated lipolysis, for removing excess hepatic LDs. Portions of LDs are recognized by the autophagic machinery, delivered through LC3-positive autophagosomes to lysosomes, and hydrolyzed by lysosomal acid lipase (LIPA), releasing fatty acids for oxidation [56,57,60]. AMPK activation, mTOR inhibition, and TFEB-dependent lysosomal biogenesis can support this response during energy stress [57,82,83]. In laying hens, induced molting has been associated with activation of an AMPK–lipophagy–lipid-metabolism pathway in the jejunum [58], providing avian evidence for lipophagy-related remodeling but not direct evidence of hepatic LD clearance. Static increases in LC3-II or autophagosome number do not by themselves prove enhanced LD clearance. Poultry studies should measure lipophagic flux using lysosomal blockade, LC3–LD colocalization, LIPA activity, and changes in LD turnover before and after pathway inhibition [57,60].

Multi-omics studies indicate that FLHS is accompanied by broad hepatic metabolic remodeling rather than simple TAG accumulation [21,22,23]. In addition, quantitative lipidomic analysis of egg yolk from FLHS laying hens revealed altered lipid composition and antioxidant capacity [78], suggesting that hepatic lipid dysregulation affects reproductive lipid allocation beyond the liver. These changes are highly relevant to LD biology because LDs not only store TAGs but also interact with membrane lipid metabolism, phospholipid remodeling, and lipid signaling pathways [14,18]. Therefore, future studies should examine LD lipid composition, LD surface proteome, and LD–organelle contact sites in poultry fatty liver, rather than focusing solely on total hepatic TG content.

Taken together, LDs may have dual implications in poultry fatty liver. On the one hand, LD formation may protect hepatocytes by buffering excess fatty acids in a relatively inert TAG pool [20]. On the other hand, persistent LD expansion, impaired LD turnover, defective LD–mitochondria coordination, oxidative stress, and inflammatory activation may accompany or contribute to liver injury and FLHS progression [72]. Thus, restoring LD homeostasis may represent a promising strategy for preventing or alleviating FLHS in poultry, but future studies should distinguish descriptive LD accumulation from mechanistic causality. The transition from adaptive hepatic LD storage to pathological overload, together with the distinct intramyocellular and intramuscular-adipocyte lipid compartments, is summarized in Figure 2.

### 3.2. LDs in Muscle Lipid Deposition and Meat Quality

Intramuscular fat (IMF) is an important determinant of poultry meat quality. Although chicken meat does not exhibit visible marbling to the same extent as beef or pork, IMF content is associated with flavor, tenderness, juiciness, water-holding capacity, and consumer acceptance [54,84,85,86]. However, this relationship is not simply linear. Improving meat quality requires optimizing IMF deposition, lipid composition, and muscle metabolic status while avoiding excessive abdominal fat accumulation or excessive intramuscular lipid deposition [84,86,87,88,89,90]. Recent selected-line, transcriptomic, lipidomic, metabolomic, and multi-omics studies have shown that IMF deposition in poultry muscle is associated with triglyceride accumulation, phospholipid remodeling, fatty acid composition, lipid uptake, adipogenic differentiation, glycerolipid metabolism, PPAR signaling, extracellular-matrix remodeling, and broader metabolic pathway reprogramming [91,92,93,94,95,96,97,98].

#### 3.2.1. Intramyocellular LDs Versus Intramuscular Adipocytes

The analytical term intramuscular fat combines at least two biologically distinct lipid compartments [99]. Intramyocellular LDs are small neutral-lipid organelles located inside myofibers, often close to mitochondria, and are regulated by myocyte lipid uptake, PLIN proteins, lipolysis, and oxidative demand [49,50,100]. In contrast, adipocytes located within intramuscular connective tissue store TAG in large unilocular LDs and are governed primarily by adipocyte recruitment, differentiation, hypertrophy, and extracellular-matrix organization [94,95]. Chemical extraction of total muscle lipid does not distinguish these pools [99]. Consequently, an increase in IMF may reflect more myocellular LDs, expansion of interstitial adipocytes, or both, and the two mechanisms should be evaluated separately by histology, cell-type markers, and spatial lipid imaging.

In skeletal muscle, LDs are the major intracellular structures storing neutral lipids. Unlike large unilocular LDs in adipocytes, muscle LDs are generally smaller, more heterogeneous, and often distributed near mitochondria, particularly in oxidative fibers [49,50,51,52]. This spatial organization may support coupling between lipid storage and mitochondrial fatty acid oxidation, allowing fatty acids released from LDs to be mobilized for energy production [100,101,102,103]. Therefore, muscle LDs should not be viewed merely as inert ectopic fat deposits; instead, they are metabolically active organelles involved in energy buffering, lipid flux control, redox balance, and cellular adaptation [100,103,104]. Whether LD accumulation improves meat quality or causes metabolic stress likely depends on LD size, lipid composition, turnover, mitochondrial coupling, and the overall metabolic state of muscle [103,104,105,106,107].

The relationship between IMF and meat quality depends not only on total lipid content but also on lipid class, fatty acid composition, cellular localization, and the degree of lipid accumulation. A selected population study in chickens demonstrated that increased IMF was associated with improved flavor and tenderness, and that IMF content was largely determined by triglycerides and phospholipids in muscle tissue, with triglycerides showing a particularly decisive contribution to IMF deposition [85]. Divergent-selection and lipidomic studies further indicate that differences in IMF deposition are accompanied by remodeling of glycerolipids, phospholipids, unsaturated fatty acids, and other lipid species in poultry muscle [93,97]. These findings are relevant to LD biology because TAGs are primarily stored within LDs, whereas phospholipids constitute cellular membranes and the LD surface monolayer [49,50]. Therefore, LD formation, TAG storage, and phospholipid remodeling may jointly influence muscle lipid deposition, fatty acid availability, and meat quality formation [91,93,97].

At the molecular level, muscle lipid deposition is regulated by lipid uptake, fatty acid activation, TAG synthesis, LD stabilization, adipogenic differentiation, and fatty acid oxidation. Candidate genes and pathways involved in chicken IMF deposition include *CD36*, *LPL*, *FASN*, *SCD*, *DGATs*, PPAR signaling, glycerolipid metabolism, steroid biosynthesis, glycerophospholipid metabolism, and carbohydrate metabolism [94,95,96,97,98]. Transcriptomic analyses of broiler pectoralis muscle with divergent TG content identified adipogenesis- and lipogenesis-related genes such as *ADIPOQ*, *PLIN1*, *CIDEC*, and *PPARG* as candidate regulators of muscle lipid accumulation [54]. Studies comparing intramuscular and abdominal fat-derived preadipocytes further showed that these two fat depots differ in adipogenic capacity and lipid metabolic programming, suggesting that IMF deposition is regulated by depot-specific cellular and molecular mechanisms [94,95]. However, most current poultry studies have focused on transcriptomic, genomic, epigenomic, lipidomic, or metabolomic associations, whereas direct evidence linking LD dynamics to poultry muscle lipid deposition remains limited. Future studies should investigate whether LD-associated proteins such as PLIN2, PLIN5, PLIN1, and CIDE family members regulate IMF accumulation by altering LD size, number, stability, lipid composition, and organelle interactions.

#### 3.2.2. Pectoralis Major Versus Thigh Muscle

LD accumulation also differs according to muscle-fiber metabolism. The chicken pectoralis major is dominated by fast glycolytic fibers, has relatively low mitochondrial density and oxidative capacity, and generally relies less on sustained fatty-acid oxidation. Thigh muscles contain a higher proportion of oxidative fibers, greater mitochondrial abundance, and stronger capacity for fatty-acid utilization [7]. These differences predict fewer or less oxidation-coupled intramyocellular LDs in breast muscle and more extensive LD–mitochondria coupling in thigh muscle, although direct quantitative imaging in poultry remains limited. Comparisons between breast and thigh should therefore control for fiber type and should not interpret tissue-level TG differences as equivalent LD biology.

LD–mitochondria coupling may be particularly important for muscle lipid metabolism. In mammalian oxidative tissues, PLIN5 has been implicated in coordinating LD storage with mitochondrial fatty acid oxidation and protecting cells from excessive fatty acid flux [49,50,51,52]. PLIN5 can localize not only to LDs but also near mitochondria, suggesting a potential role in facilitating LD–mitochondria communication and LD-derived fatty acid oxidation [49,50]. In skeletal muscle models, PLIN5 overexpression increases intramyocellular lipid storage while promoting oxidative gene expression and maintaining insulin sensitivity, indicating that LD accumulation can be metabolically adaptive when coupled to mitochondrial oxidative capacity [51,52]. Conversely, impaired coordination between lipid storage and oxidation may promote lipotoxic stress, mitochondrial dysfunction, and reduced muscle metabolic flexibility [103]. Although these mechanisms have been mainly studied in mammals, they provide a useful hypothesis for poultry muscle lipid metabolism. In chicken skeletal muscle, whether LD–mitochondria coupling directly affects postmortem biochemical processes and meat quality remains to be tested.

The distinction between beneficial IMF deposition and excessive lipid accumulation is important. Moderate and properly regulated LD storage in muscle may support desirable flavor, tenderness, and juiciness by contributing to TAG and fatty acid pools [84,85]. However, excessive lipid deposition, abnormal LD enlargement, unfavorable fatty acid composition, or impaired LD turnover may disrupt mitochondrial function, increase oxidative deterioration, and negatively affect muscle physiology and product quality [103]. Therefore, the goal of poultry lipid regulation should not be simply to increase lipid storage but to optimize LD homeostasis, including LD formation, lipid composition, degradation, spatial distribution, and mitochondrial coupling.

#### 3.2.3. Post-Mortem Lipid Oxidation and Product Stability

After slaughter, ATP depletion terminates controlled LD turnover and mitochondrial fatty-acid utilization, while neutral lipids and, particularly, PUFA-rich membrane phospholipids become substrates for nonenzymatic oxidation [108]. Hydroperoxides and secondary aldehydes generate rancid or warmed-over flavors and can reduce consumer acceptability during refrigerated or frozen storage [108]. Lipid-derived radicals also promote myoglobin oxidation, linking lipid oxidation to discoloration and reduced color stability [108]. Thigh meat may be especially vulnerable because of its higher myoglobin content, mitochondrial abundance, and oxidative lipid environment, whereas breast meat has a different balance of lipid amount and antioxidant protection [7,108]. Whether pre-slaughter LD size, phospholipid monolayer composition, or LD–mitochondria organization directly predicts post-mortem rancidity remains unresolved and should be tested using spatial lipidomics together with TBARS, volatile aldehydes, and color measurements.

In summary, LDs provide a conceptual bridge between muscle lipid deposition and meat quality. They may influence how fatty acids are stored, mobilized, and oxidized in muscle cells. A better understanding of LD dynamics in poultry skeletal muscle may help develop strategies to optimize IMF deposition while reducing undesirable abdominal fat accumulation [84,86,89,90]. This perspective may be useful for integrating genetic selection, nutritional regulation, adipocyte biology, muscle metabolism, and meat quality improvement in poultry production, provided that future studies validate LD mechanisms directly in poultry tissues.

## 4. LD–Organelle Interactions: Mechanistic Insights from Model Systems and Potential Relevance to Poultry Lipid Metabolism

LDs are increasingly recognized as dynamic organelles that communicate with other cellular compartments [109,110,111,112,113]. These physical and functional interactions, commonly referred to as membrane contact sites, enable the exchange of lipids, proteins, metabolites, and signals without complete organelle fusion [109,110,111,112,113]. In lipid-metabolically active tissues such as liver and skeletal muscle, LD–organelle interactions may influence whether fatty acids are stored in neutral lipid pools, oxidized for energy production, incorporated into membranes, or converted into lipotoxic intermediates [49,50,100,102,103,104]. However, most mechanistic studies have been performed in mammals, yeast, and invertebrate models. Therefore, their relevance to poultry should be considered as a hypothesis that requires direct validation in avian tissues.

In poultry, LD–organelle interactions may provide a mechanistic framework for studying fatty liver, intramuscular lipid deposition, heat stress-related metabolic injury, and nutritional regulation. For example, LD–mitochondria coupling may influence whether muscle lipids are retained as IMF or mobilized for oxidation, whereas LD–ER and LD–peroxisome interactions may regulate TAG synthesis, phospholipid remodeling, and fatty acid chain modification [109,110,111,112,113]. However, direct evidence in avian species remains limited. Therefore, integrating LD contact-site biology into poultry lipid metabolism should be presented as a way to generate testable hypotheses rather than as established causality. The protein-defined LD–ER, LD–mitochondria, LD–peroxisome, and LD–lysosome contact sites discussed below are summarized in Figure 3.

### 4.1. LD–ER Interactions: Coordinating Lipid Synthesis and LD Biogenesis

The endoplasmic reticulum (ER) is the major site of LD biogenesis. Neutral lipids, mainly triacylglycerols and sterol esters, are synthesized within the ER membrane and accumulate between the two leaflets of the ER bilayer. Once a critical concentration is reached, these neutral lipids nucleate to form lipid lenses that bud toward the cytoplasm and mature into LDs [13,114,115]. Thus, the LD–ER interface is not only the birthplace of LDs but also a regulatory platform controlling LD number, size, lipid composition, and protein targeting [37,112,116].

Several proteins are involved in LD formation at ER–LD contact sites. Seipin plays an important role in LD nucleation and maturation by organizing sites of neutral lipid accumulation and stabilizing nascent LDs [34,37,117]. DGAT2, a key enzyme catalyzing the final step of triacylglycerol synthesis, can localize to ER–LD interfaces, supporting local TAG production for LD expansion [33,70,118]. In addition, ER-resident and LD-associated proteins may coordinate phospholipid supply, surface tension, and LD growth [112,116,119]. These mechanisms are particularly relevant to avian hepatocytes, where hepatic lipogenesis is highly active and newly synthesized lipids must be efficiently stored, remodeled, or exported [1,3].

ER–LD contacts also require defined molecular scaffolds. VAP-A and VAP-B are ER-resident proteins that recruit FFAT-motif-containing lipid-transfer factors, including oxysterol-binding protein-related proteins, and thereby organize lipid exchange at membrane contact sites. The related ER protein MOSPD2 can bind LDs through its CRAL-TRIO domain and acts as a direct ER–LD tether in mammalian cells [120]. Together with seipin and DGAT2, VAP-family complexes may coordinate phospholipid delivery, LD protein targeting, and continued TAG synthesis after LD budding. These mechanisms provide specific candidates for avian hepatocytes, but VAP-A/B- or MOSPD2-dependent ER–LD tethering has not yet been established in poultry.

In poultry fatty liver, excessive hepatic lipid synthesis may place a high burden on the ER–LD system. When TAG production exceeds the capacity of LD maturation, VLDL assembly, or lipid export, enlarged LDs accumulate within hepatocytes [4,21,64]. At the same time, persistent lipid overload may disturb ER homeostasis and induce ER stress. ER stress can further disrupt lipid metabolism by altering lipogenic gene expression, inflammatory signaling, and protein folding capacity [74,75]. Therefore, LD–ER dysfunction may represent an early event linking hepatic lipid overload to fatty liver progression.

The LD–ER axis also has implications for nutritional regulation. Dietary energy level, fatty acid composition, choline, methionine, and other methyl donors may influence hepatic TAG synthesis, phosphatidylcholine availability, VLDL secretion, and LD surface stability [25,64,71]. In laying hens, where hepatic lipid export to the ovary is strongly activated during egg production, maintaining ER–LD homeostasis is likely essential for both reproductive performance and liver health [3,64]. Future studies should investigate how ER–LD contact-site proteins and lipid synthetic enzymes respond to high-energy diets, heat stress, and nutritional interventions in poultry.

### 4.2. LD–Mitochondria Interactions: Linking Lipid Storage to Fatty Acid Oxidation

Mitochondria are central organelles for fatty acid β-oxidation, ATP production, redox balance, and metabolic signaling. LD–mitochondria contacts provide a spatial mechanism by which fatty acids stored in LDs can be mobilized and transferred to mitochondria for oxidation [50,109,121]. This coupling is especially important in tissues with high oxidative capacity, including skeletal muscle, heart, and liver [49,51,52]. Studies in mammalian systems indicate that LD–mitochondria contacts are dynamically regulated by nutritional status, exercise, fasting, cold exposure, and cellular energy demand [102,121,122].

PLIN5 is one of the best-characterized LD-associated proteins involved in LD–mitochondria coupling. PLIN5 is enriched in oxidative tissues and has been proposed, mainly in mammalian models, to coordinate lipid storage with mitochondrial fatty acid oxidation [49,50,51]. It can localize at LD surfaces and has also been observed near mitochondria, supporting its role in connecting LDs with oxidative metabolism [49,50]. Recent studies have further shown that PLIN5 can interact with FATP4 at membrane contact sites and promote LD-to-mitochondria fatty acid transport under starvation conditions [53]. In this model, LD–mitochondria coupling does not merely bring two organelles together; rather, it helps channel fatty acids toward mitochondrial oxidation while limiting uncontrolled lipotoxicity [53,102]. Whether poultry PLIN5 performs similar contact-site functions remains an important question.

Mitofusin-2 (MFN2) should be considered a candidate regulator rather than an established poultry LD–mitochondria tether. In mammalian cardiomyocytes, an MFN2-Hsc70 complex has been reported to form mitochondria–LD contacts and regulate lipid use, while exercise-induced MFN2 altered hepatic LD–mitochondria communication and promoted fatty-acid oxidation in mouse NAFLD models [123,124]. MFN2 may therefore influence contact stability, mitochondrial fusion, and oxidative capacity simultaneously. Poultry-specific loss-of-function experiments are required to separate a direct tethering role from indirect effects on mitochondrial morphology and metabolism.

Importantly, LD–mitochondria proximity does not have a single metabolic meaning. Oxidative contacts may combine ATGL-dependent fatty-acid release, PLIN5–FATP4-mediated transfer, CPT1A entry, and β-oxidation to meet energy demand [53]. Other peridroplet mitochondria preferentially provide ATP and biosynthetic substrates for TAG synthesis and LD expansion, supporting lipid sequestration rather than immediate oxidation [122]. Contact formation can therefore either channel fatty acids toward energy production or contain excess fatty acids in a less toxic TAG pool. Increased contact frequency alone should not be interpreted as increased β-oxidation without parallel fatty-acid tracing, oxygen-consumption, and lipolytic-flux measurements.

For poultry, LD–mitochondria interactions may be important in both liver and skeletal muscle. In the liver, efficient coordination between LD storage and mitochondrial oxidation may help reduce excessive lipid accumulation. Conversely, defective mitochondrial oxidation may be associated with LD expansion and hepatic steatosis [19,73]. This is relevant to fatty liver hemorrhagic syndrome, in which lipid accumulation is frequently accompanied by oxidative stress and mitochondrial dysfunction [21,22,73]. In skeletal muscle, LD–mitochondria contacts may influence fatty acid utilization, muscle energy metabolism, and meat quality. Moderate intramuscular lipid deposition may improve flavor and tenderness within an appropriate range, but this lipid pool must be metabolically coordinated with mitochondrial oxidative capacity to avoid oxidative damage [49,84,85].

LD–mitochondria interactions may also contribute to stress adaptation in poultry. Under heat stress, mitochondrial dysfunction and ROS production are common features of metabolic injury [2,125,126]. LDs may buffer excess fatty acids and provide controlled lipid substrates to mitochondria, thereby reducing lipotoxic stress [20,103]. However, if LD–mitochondria coupling is impaired, fatty acids may accumulate as toxic intermediates or overload mitochondrial oxidation, promoting oxidative damage [103,122]. Therefore, LD–mitochondria communication may represent a potential node connecting lipid metabolism, oxidative stress, and stress tolerance in poultry, but direct avian evidence is still needed.

Despite its potential importance, direct evidence for LD–mitochondria contact regulation in poultry remains scarce. Future studies should combine confocal microscopy, transmission electron microscopy, super-resolution imaging, organelle isolation, and lipid tracing to determine whether LD–mitochondria contacts are altered in fatty liver, muscle lipid deposition, heat stress, or nutritional interventions [100,109,127]. Particular attention should be paid to poultry PLIN5, mitochondrial fatty acid transport proteins, CPT1A, PPARα signaling, and AMPK-mediated metabolic regulation [53,82,102].

### 4.3. LD–Peroxisome Interactions: Connecting Lipid Turnover, VLCFA Metabolism and Oxidative Balance

Peroxisomes are essential organelles involved in very-long-chain fatty acid oxidation, branched-chain fatty acid metabolism, ether lipid synthesis, and reactive oxygen species homeostasis [128,129]. They are metabolically connected to both LDs and mitochondria, forming an integrated network for lipid handling and redox regulation [110,130]. LD–peroxisome interactions have been observed in several biological systems and are thought to facilitate fatty acid trafficking from LDs to peroxisomes for β-oxidation or lipid remodeling [17,131].

One important feature of peroxisomes is their ability to oxidize fatty acids that are poor substrates for mitochondria, especially very-long-chain fatty acids [128,129]. Through LD–peroxisome contacts, fatty acids released from LDs may be directed to peroxisomes for shortening, after which products can be further metabolized by mitochondria [130,132]. This peroxisome–mitochondria metabolic cooperation may be particularly important under lipid overload conditions. In addition, peroxisomes contribute to the synthesis of ether lipids, including plasmalogens, which are important components of membranes and may protect cells against oxidative stress [128,133].

Mechanistic studies have identified proteins that can mediate LD–peroxisome contact and fatty acid transfer. For example, spastin has been shown to tether LDs to peroxisomes through ABCD1 and promote fatty acid trafficking through ESCRT-III-dependent mechanisms [134]. Other studies have revealed dynamic spatial contacts between LDs and peroxisomes that support lipid mobilization and metabolic adaptation [110,131]. These findings are not yet well established in poultry and should therefore be treated as hypotheses for future avian studies rather than as poultry-specific mechanisms.

In poultry liver, LD–peroxisome interactions may be relevant to fatty liver development and oxidative stress. When hepatocytes accumulate large amounts of lipids, peroxisomal β-oxidation may help process specific fatty acid species and reduce lipid burden [128,129]. However, peroxisomal oxidation also produces hydrogen peroxide, making antioxidant capacity critical for maintaining redox balance [129,135]. If peroxisomal lipid oxidation and antioxidant systems become uncoupled, LD–peroxisome interactions may contribute to oxidative injury rather than protection. This mechanism may be particularly relevant under high-energy feeding and fatty liver conditions [64], whereas its relationship with heat stress requires separate validation.

In skeletal muscle, LD–peroxisome interactions remain even less understood but may contribute to the handling of complex fatty acid species and oxidative balance [17,131]. Given that poultry production is often challenged by rapid growth, high metabolic demand, and environmental stress, peroxisome-related lipid metabolism may deserve more attention [5,66,125]. Future studies should examine peroxisomal markers such as PMP70, ACOX1, catalase, PEX proteins, and their spatial relationship with LDs in poultry liver and muscle. The LD–peroxisome axis may represent an underexplored mechanism linking lipid turnover, oxidative stress, and metabolic resilience in poultry.

### 4.4. LD–Lysosome Interactions and Lipophagy: Degradation of LDs Through Autophagy

In addition to cytosolic lipolysis, LDs can be degraded through lysosome-dependent autophagy, a process known as lipophagy. During lipophagy, LDs or portions of LDs are engulfed by autophagosomes and delivered to lysosomes, where neutral lipids are hydrolyzed by lysosomal acid lipase [56,61,83]. Lipophagy provides fatty acids for energy production and contributes to the clearance of excessive LDs, especially in hepatocytes [57,60].

Lipophagy is closely linked to nutritional status. Under fasting or energy deprivation, activation of autophagy promotes LD breakdown and fatty acid mobilization, thereby supporting mitochondrial β-oxidation [83,121]. Conversely, impaired autophagy can lead to excessive hepatic lipid accumulation and metabolic dysfunction in mammalian models [56,61]. Since poultry liver is the central organ for de novo lipogenesis and lipid export, lipophagy may be an important mechanism protecting against fatty liver [1,64]. However, compared with mammalian NAFLD models, avian lipophagy remains poorly investigated.

In laying hens, impaired lipophagy may contribute to FLHS by limiting LD clearance under conditions of high lipid synthesis, but direct hepatic flux evidence is currently lacking. A recent laying-hen study showed that induced molting activates an AMPK–lipophagy–lipid-metabolism pathway in the jejunum, not the liver [58], supporting a broader avian role for lipophagy but not directly establishing hepatic lipophagic flux. In broilers, altered autophagy may influence fat deposition and metabolic efficiency. Under heat stress, autophagy may have dual roles: it can remove damaged organelles and mobilize lipids, but excessive or dysregulated autophagy may also contribute to tissue injury [125,136]. Therefore, the role of lipophagy in poultry lipid metabolism likely depends on tissue type, nutritional status, stress intensity, and production stage.

Several nutritional interventions may influence lipophagy. Functional nutrients and phytochemicals that activate AMPK, inhibit mTOR, improve mitochondrial function, or reduce oxidative stress may enhance LD turnover and alleviate lipid accumulation [57,82,137]. For example, resveratrol, curcumin, polyphenols, and certain probiotics have been proposed to regulate lipid metabolism partly through autophagy-related pathways in animal models [137,138]. Future poultry studies should determine whether these interventions directly modulate LD–lysosome interactions and lipophagic flux rather than only measuring total hepatic lipid content.

Overall, LD–lysosome interactions represent an important but underexplored mechanism in poultry lipid metabolism. Establishing reliable methods to monitor lipophagy in avian tissues, including LC3 colocalization with LDs, lysosomal acid lipase activity, autophagic flux assays, and ultrastructural analysis, will be necessary to clarify the contribution of lipophagy to fatty liver prevention and metabolic adaptation [57,60,83].

### 4.5. Summary: Organelle Interactions as a Potential LD-Associated Metabolic Network

LD–organelle interactions provide a useful framework for understanding poultry lipid metabolism beyond the traditional focus on lipid synthesis and fat deposition. LD–ER contacts regulate lipid synthesis and LD formation; LD–mitochondria contacts coordinate fatty acid oxidation and energy metabolism; LD–peroxisome contacts contribute to very-long-chain fatty acid metabolism and redox balance; and LD–lysosome interactions mediate lipophagic degradation and lipid clearance [17,109,111]. However, for poultry, many of these contact-site mechanisms are still inferred from non-avian models and require direct experimental validation.

For poultry, this potential LD-associated organelle network may be relevant to several production-related traits and disorders, including fatty liver, abdominal fat deposition, intramuscular fat accumulation, meat quality, egg production, and heat stress tolerance [2,64,99]. However, most evidence remains indirect, and poultry-specific mechanistic studies are urgently needed. Future research should move from descriptive measurements of lipid content toward spatial, dynamic, and causal analyses of LD function. By integrating imaging, lipidomics, proteomics, gene editing, and nutritional intervention approaches, LD–organelle interactions may become promising hypotheses and potential targets for improving poultry metabolic health and production performance [9,78,109].

## 5. Nutritional Regulation of LD Metabolism in Poultry

Nutritional regulation is one of the most practical strategies for modulating lipid metabolism in poultry. In practical poultry diets, corn/maize and soybean meal are major sources of dietary energy, protein, and amino acids, while supplemental oils, amino acids, vitamins, minerals, and functional additives are used to adjust nutrient supply. Because the avian liver is the predominant site of de novo lipogenesis, dietary energy level, fatty acid composition, amino acid balance, choline and methionine supply, antioxidants, trace elements, phytochemicals, and gut microbiota-modulating additives can all influence hepatic lipid synthesis, lipid export, LD formation, and lipid turnover [1,25,64]. Nutritional interventions may alleviate excessive hepatic lipid accumulation by reducing lipogenesis, promoting very low-density lipoprotein (VLDL) secretion, enhancing fatty acid oxidation, stimulating LD degradation, improving mitochondrial function, and limiting oxidative stress [65,73,139]. Recent poultry studies and reviews have increasingly emphasized nutritional strategies that target AMPK, PPARα, oxidative stress, mitochondrial homeostasis, and lipid transport pathways in fatty liver and lipid metabolic disorders [126,139,140]. The dietary categories, molecular targets, and physiological or adverse outcomes discussed in this section are summarized in Figure 4.

### 5.1. Dietary Energy Level and Fatty Acid Composition

Dietary energy level is a major determinant of lipid deposition in poultry. Corn/maize-based energy supply, dietary oil inclusion, and the energy-to-protein ratio can influence hepatic de novo lipogenesis and the availability of fatty acids for triacylglycerol (TAG) synthesis and LD expansion. It should be noted that higher dietary energy density in poultry diets is commonly achieved by increasing oil or fat inclusion. Consequently, effects attributed to dietary energy may be confounded by fat inclusion level, lipid source, and fatty acid composition; appropriately controlled or factorial dietary designs are therefore needed to separate energy-density effects from those of dietary lipid supply. When hepatic lipid synthesis exceeds the capacity for fatty acid oxidation and VLDL secretion, TAG-rich LDs accumulate in hepatocytes, increasing the risk of fatty liver and fatty liver hemorrhagic syndrome [4,64,139]. Conversely, appropriate energy restriction or balanced energy-to-protein ratio may reduce excessive fat deposition and improve metabolic health. Therefore, controlling dietary energy supply is a fundamental nutritional strategy for maintaining LD-associated lipid homeostasis in poultry [65,139].

Fatty acid composition also has a strong influence on LD biology. Saturated fatty acids are more likely to induce lipotoxicity, endoplasmic reticulum stress, mitochondrial dysfunction, and inflammatory responses when present in excess [20,103]. In contrast, unsaturated fatty acids, especially monounsaturated fatty acids and n-3 polyunsaturated fatty acids (PUFAs), may promote safer storage of fatty acids as TAGs, improve membrane lipid composition, enhance fatty acid oxidation, and reduce inflammatory signaling [141,142]. In poultry, dietary flaxseed, fish oil, linseed oil, and other n-3 PUFA-rich ingredients have been widely studied for their effects on egg lipid composition, tissue fatty acid profile, and lipid metabolism. Several studies have shown that dietary n-3 fatty acids can alter hepatic lipid composition and egg yolk fatty acid enrichment, indicating that dietary fatty acids are efficiently incorporated into avian lipid metabolic pathways [141,142].

From an LD perspective, dietary fatty acids may affect not only total lipid accumulation but also LD composition and function. TAG species stored in LDs determine the physical properties of LDs, lipase accessibility, and the susceptibility of cells to oxidative stress [114,115]. In hepatocytes, enrichment of unsaturated fatty acids may facilitate TAG formation and reduce free fatty acid toxicity, whereas excessive saturated fatty acids may overwhelm LD buffering capacity and promote lipotoxic injury [20,141]. In skeletal muscle, fatty acid composition may influence intramuscular lipid deposition, mitochondrial oxidation, and meat flavor [84,85]. Thus, future poultry studies should evaluate how different fatty acid sources regulate LD size, LD number, LD lipid composition, and LD–mitochondria coupling, rather than focusing only on total fat deposition.

### 5.2. Lipotropic Nutrients and Methyl Donor Metabolism: Choline and Methionine

Lipotropic nutrients and methyl donor metabolism are particularly important for poultry liver health because they support phospholipid synthesis, VLDL assembly, methyl metabolism, antioxidant defense, and lipid export [71,143]. Choline is a precursor for phosphatidylcholine, a major phospholipid required for VLDL formation and secretion. Inadequate choline supply can impair hepatic lipid export, resulting in TAG retention and LD accumulation in hepatocytes. Therefore, choline supplementation has long been considered a nutritional approach to prevent or reduce fatty liver in poultry [71,143].

Betaine, an oxidized derivative of choline, functions as an osmoprotectant and methyl donor. It should be described as a functional methyl donor or specialized feed additive that has been examined in specific nutritional contexts, rather than as a universal basal feed additive. Recent studies have shown that betaine supplementation can alleviate early fatty liver in laying hens by improving lipid metabolism and reducing oxidative stress, suggesting that methyl donor status is closely associated with hepatic LD homeostasis [140,144]. Mechanistically, betaine may support hepatic lipid metabolism through several routes: improving methyl group availability, enhancing phosphatidylcholine synthesis, promoting lipid export, regulating antioxidant capacity, and modulating genes involved in lipogenesis and fatty acid oxidation [140,144]. These effects are likely to reduce excessive LD accumulation and protect hepatocytes from lipid-induced damage.

Methionine is another essential methyl donor and sulfur-containing amino acid involved in one-carbon metabolism, glutathione synthesis, and antioxidant defense. Appropriate methionine supply is necessary for protein synthesis, hepatic function, and lipid metabolism in poultry. Recent work suggests that methionine source, sulfur amino acid balance, and vitamin E status can influence antioxidant potential and hepatic oxidative status in aged laying hens [145]. Since oxidative stress is closely associated with LD dysfunction and fatty liver progression, methionine may regulate LD metabolism not only through methyl donation but also through redox control [2,145].

From an LD-associated lipid handling viewpoint, choline and methionine are core nutrients supporting the balance between LD storage and lipid export, whereas betaine should be discussed as a functional methyl donor evaluated in specific studies. When VLDL assembly and phospholipid availability are sufficient, TAGs can be exported from the liver rather than retained as enlarged LDs [3,71]. Conversely, inadequate methyl donor or lipotropic nutrient supply may promote hepatic LD accumulation and increase susceptibility to fatty liver [140,143]. Therefore, choline and methionine nutrition should be considered key components of nutritional strategies for poultry liver health.

### 5.3. Vitamins, Trace Elements and Antioxidant Nutrients

Oxidative stress is a major contributor to lipid metabolic disorders in poultry. Excessive hepatic lipid accumulation increases mitochondrial and peroxisomal fatty acid oxidation, which can elevate reactive oxygen species production [2,126]. At the same time, lipid peroxidation can damage LD-associated membranes, mitochondrial membranes, and cellular proteins, thereby worsening metabolic dysfunction [103,126]. Antioxidant nutrients can therefore influence LD homeostasis indirectly by protecting mitochondria, maintaining redox balance, and preventing lipid peroxidation.

Vitamin E is one of the most important lipid-soluble antioxidants in poultry nutrition. It protects cellular membranes and lipids from oxidative damage and may help maintain hepatic and muscle lipid stability [145]. In laying hens, vitamin E is often studied together with methionine or other antioxidant nutrients because sulfur amino acid metabolism and antioxidant defense are closely connected [145]. By reducing oxidative stress, vitamin E may help prevent the transition from adaptive LD storage to pathological lipid-induced injury.

Selenium is another important trace element involved in antioxidant defense, mainly through its incorporation into selenoproteins such as glutathione peroxidases and thioredoxin reductases. Selenium supplementation has been widely studied in poultry for its effects on antioxidant capacity, immune function, reproductive performance, and meat quality [146,147]. In the context of LD metabolism, selenium may protect cells from lipid peroxidation and mitochondrial oxidative damage, thereby helping maintain LD–mitochondria metabolic coupling [147,148]. Although direct evidence linking selenium to LD dynamics in poultry remains limited, selenium-regulated redox pathways may influence LD stability, fatty acid oxidation, and stress adaptation.

Other trace elements, including zinc, chromium, manganese, and copper, may also affect lipid metabolism through insulin signaling, antioxidant enzymes, mitochondrial function, and inflammatory responses [148,149]. However, their direct roles in LD formation, LD degradation, and LD–organelle interactions remain insufficiently understood. Future studies should clarify whether antioxidant nutrients regulate poultry lipid metabolism by altering LD-associated protein expression, LD lipid composition, and organelle contact sites.

### 5.4. Phytochemicals and Functional Plant-Derived Additives

Plant-derived bioactive compounds have attracted increasing interest as nutritional modulators of lipid metabolism in poultry. Polyphenols, flavonoids, curcumin, resveratrol, tea polyphenols, and plant extracts can influence lipid metabolism through antioxidant, anti-inflammatory, gut microbiota-modulating, and metabolic signaling pathways [149,150,151]. A study of quercetin in broilers showed that this flavonoid improved lipid metabolism through gut microbiota remodeling and AMPK/PPAR signaling, supporting the potential of dietary flavonoids to regulate lipid deposition and metabolic homeostasis in poultry [149].

Curcumin, the major bioactive compound of turmeric, has been reported to regulate lipid metabolism and antioxidant capacity in poultry. Studies in laying hens indicate that curcumin can improve antioxidant status under thermal stress and modulate hepatic lipid metabolism-related pathways [150,151]. From an LD-associated perspective, curcumin and related compounds may reduce pathological LD accumulation by suppressing lipogenesis, activating AMPK-related pathways, improving mitochondrial function, and reducing oxidative stress.

Resveratrol and other polyphenols may also regulate LD metabolism by activating AMPK and PPARα, inhibiting SREBP1c-mediated lipogenesis, improving mitochondrial oxidative capacity, and enhancing autophagy [82,138]. Although many of these mechanisms have been established mainly in mammalian models, poultry studies suggest that polyphenol-rich additives can improve lipid metabolism and oxidative status [149,150,151]. For LD biology, the most important question is whether these additives directly affect LD size, LD number, lipolysis, lipophagy, or LD–mitochondria interactions in poultry tissues. Where the cited poultry experiments measured only hepatic TG, histology, AMPK/PPAR signaling, or transcriptional changes, these findings should be interpreted as indirect evidence of altered lipid metabolism rather than proof that a compound changed LD size, lipase recruitment, lipophagic flux, or LD–organelle contacts.

Marigold extract, tea polyphenols, plant essential oils, and other functional plant products may also contribute to poultry lipid metabolic health. For example, recent studies in late-laying hens have linked plant-derived antioxidant additives with improved lipid metabolism, antioxidant capacity, and microbial composition [149,152,153]. These findings suggest that phytochemicals may regulate LD metabolism through both direct hepatic effects and indirect gut microbiota-related mechanisms.

### 5.5. Probiotics, Prebiotics and Gut Microbiota-Derived Signals

The gut microbiota is increasingly recognized as an important regulator of host lipid metabolism. In poultry, intestinal microbes can influence nutrient digestion, bile acid metabolism, short-chain fatty acid production, intestinal barrier integrity, systemic inflammation, and hepatic lipid metabolism [154,155]. Therefore, probiotics, prebiotics, synbiotics, and postbiotics may regulate hepatic LD accumulation indirectly through the gut–liver axis.

Gut microbiota-derived metabolites, including short-chain fatty acids and indole derivatives, together with microbially modified bile acids, can modulate host lipid metabolism by regulating intestinal nutrient absorption, hepatic lipogenesis, fatty acid oxidation, and inflammatory signaling [154,155]. Primary bile acids are synthesized from cholesterol in the liver, whereas intestinal microorganisms deconjugate and biotransform host-derived bile acids, thereby generating secondary bile acids and reshaping the composition and signaling properties of the enterohepatic bile acid pool. The resulting bile acid pool is particularly relevant because bile acids function not only as lipid emulsifiers but also as signaling molecules acting through receptors such as FXR and TGR5 [155,156]. Recent poultry studies indicate that bile acid supplementation can reduce hepatic lipid accumulation and alter liver fatty acid profiles in late-phase laying hens, suggesting that bile acid signaling may be a promising target for improving liver health [156,157,158].

Probiotics may reduce lipid metabolic disorders by improving gut barrier function, decreasing endotoxin translocation, modulating bile acid metabolism, reducing inflammation, and altering energy harvest [154,155]. These effects may ultimately influence hepatic LD formation and turnover. For example, reduced systemic inflammation and improved bile acid signaling may suppress excessive hepatic lipogenesis and promote fatty acid oxidation [155,156]. In addition, certain probiotics may increase short-chain fatty acid production, which can influence AMPK activation and lipid metabolism [82,154]. Likewise, microbiota remodeling or reduced liver fat does not establish direct regulation of LD behavior unless LD-specific structural or flux endpoints are measured.

Despite these promising findings, direct links between gut microbiota and LD biology in poultry remain largely unexplored. Future studies should determine whether probiotics or microbiota-derived metabolites regulate hepatic LD size, LD lipid composition, LD-associated proteins, lipophagy, and LD–mitochondria interactions. A “gut microbiota–liver–LD axis” may provide a useful framework for understanding how nutritional additives improve poultry metabolic health [154,155].

### 5.6. Evidence Strength, Dose–Response, and Commercial Translation

Nutritional evidence should be graded according to the endpoints actually measured. Direct LD evidence includes quantitative LD number and diameter, LD lipid or proteome composition, PLIN abundance at the LD surface, ATGL recruitment, LC3–LD colocalization with flux analysis, lysosomal acid-lipase activity, and organelle-contact measurements [57,60,100]. Hepatic TG, Oil Red O area, AMPK or PPAR signaling, and DGAT or ATGL transcript abundance provide indirect molecular evidence but do not by themselves demonstrate altered LD turnover [55,58]. Changes limited to body weight, abdominal-fat percentage, serum lipids, egg production, or feed conversion are production-level evidence. Mechanistic terms such as ‘activation of lipophagy’ or ‘improved LD–mitochondria coupling’ should therefore be reserved for studies that directly test those processes.

Dose and model context also determine translational value. For example, the betaine study cited here used 8 g/kg diet during a seven-day dexamethasone-induced early fatty-liver model [140], a relatively high and short-term challenge setting that should not be equated with routine commercial supplementation. The curcumin study tested 0, 100, 200, 400, and 800 mg/kg for eight weeks and identified 200 mg/kg as the most consistently beneficial level for egg quality and hepatic LD/TG outcomes [151], illustrating that higher inclusion did not produce uniformly greater benefit. Concentrations used in isolated hepatocytes cannot be converted directly into feed-inclusion rates because absorption, metabolism, tissue exposure, and matrix interactions differ substantially in vivo.

Commercial viability requires more than biochemical efficacy. Candidate additives should be evaluated for cost per tonne of feed, active-compound standardization, bioavailability, stability during storage and pelleting, palatability, interactions with basal diet composition, regulatory status, and effects on laying rate, egg mass, feed conversion, growth, carcass yield, mortality, and product quality. Benefits observed only in pharmacological challenge models may not persist under standard production conditions. Multi-dose, multi-cycle trials with economic analysis and direct LD endpoints are therefore needed before an intervention can be described as a practical LD-targeted strategy [25,140,145,151].

### 5.7. Summary: Toward LD-Informed Nutritional Strategies

Overall, nutritional regulation of poultry lipid metabolism can be interpreted through an LD-associated lipid handling framework. High dietary energy promotes hepatic lipogenesis and LD expansion, whereas balanced energy supply and appropriate fatty acid composition can reduce pathological lipid accumulation [64,65,139]. Choline and methionine support phospholipid synthesis, VLDL secretion, and antioxidant capacity, thereby limiting excessive hepatic LD retention [71,143,145]. Betaine may provide additional methyl-donor support in specific contexts but should not be presented as a universal feed additive [140]. Antioxidant nutrients such as vitamin E and selenium protect against lipid peroxidation and mitochondrial dysfunction [145,146,147,148]. Phytochemicals and probiotics may regulate lipid metabolism by suppressing lipogenesis, enhancing fatty acid oxidation, activating AMPK, improving mitochondrial function, promoting lipophagy, and modulating the gut–liver axis [82,149,155].

However, most poultry nutrition studies still evaluate lipid metabolism using conventional indicators such as serum triglycerides, liver fat content, abdominal fat percentage, antioxidant enzyme activity, and gene expression. Future studies should include LD-specific endpoints, such as LD size, number, lipid composition, LD-associated proteins, lipolytic activity, lipophagic flux, and LD–organelle contacts [57,60,100]. Such approaches will help identify nutritional strategies that not only reduce pathological fat accumulation but also optimize LD function for poultry health, production performance, and meat quality, while avoiding the oversimplified goal of simply increasing IMF content.

## 6. Concluding Remarks and Perspectives

LDs provide an important entry point for understanding the regulation of lipid metabolism in poultry. This review highlights that LDs are not merely lipid storage structures, but dynamic metabolic platforms involved in lipid synthesis, VLDL secretion, fatty acid oxidation, lipophagy, and organelle communication [14,29,109]. In poultry, where hepatic de novo lipogenesis predominates, hepatocellular LD homeostasis has important implications for fatty liver development, yolk lipid supply, and systemic lipid distribution [1,3,64]. Meanwhile, muscle LDs and their functional coupling with mitochondria may represent a potential link between intramuscular fat deposition and meat quality formation, but the relationship between IMF and meat quality should be understood as dose- and context-dependent rather than simply linear [49,84,85].

Future studies should move beyond traditional analyses of fat content and focus more on LD function. In particular, greater attention should be given to LD heterogeneity across tissues, the regulatory roles of LD-associated proteins, and the dynamic interactions between LDs and the endoplasmic reticulum, mitochondria, peroxisomes, and lysosomes [17,109,111]. From a nutritional perspective, it will be important to determine whether dietary energy level, fatty acid composition, choline and methionine supply, antioxidant nutrients, phytochemicals, probiotics, and functional methyl donors improve poultry metabolic health by modulating LD formation, turnover, and organelle coupling [65,140,149]. Particular attention should also be given to hepatic LD remodeling during female sexual maturation and the onset of yolk precursor synthesis, when the liver rapidly shifts toward estrogen-driven lipogenesis and VLDLy secretion [3]. Future study designs should be production-type-specific, distinguish layers from broilers, report dose–response and commercial feasibility, and combine conventional production outcomes with direct measurements of LD structure, turnover, and metabolic flux.

Overall, LD biology provides a useful but still developing framework for understanding lipid storage, mobilization, and metabolic regulation in poultry. Current evidence supports the relevance of LD-associated lipid handling to fatty liver, muscle lipid deposition, and nutritional regulation, but direct mechanistic studies in poultry remain limited. By combining high-resolution imaging, lipidomics, organelle proteomics, and functional validation, future research may establish precise nutritional and genetic strategies to regulate LD metabolism, thereby reducing pathological lipid deposition, optimizing beneficial lipid accumulation, and improving poultry health and production performance [78,100,109].

## 7. Methods

This narrative review was based on a structured search of PubMed, Web of Science Core Collection, and Scopus from database inception to 11 July 2026. Search terms combined “lipid droplet” or “lipid droplets” with poultry-related terms, including chicken, broiler, laying hen, duck, and goose, as well as fatty liver hemorrhagic syndrome, intramuscular fat, organelle interactions, and nutritional regulation.

After duplicate records were removed, titles, abstracts, and, where necessary, full texts were screened for relevance. Peer-reviewed original studies and reviews concerning lipid droplet formation, turnover, lipolysis, lipophagy, hepatic steatosis, muscle lipid deposition, organelle interactions, or nutritional regulation were included. Poultry studies were prioritized, whereas mammalian and cellular studies were included only when direct avian evidence was unavailable and were identified accordingly in the text. Conference abstracts, non-peer-reviewed reports, duplicate publications, and unrelated studies were excluded. Reference lists of relevant articles were also screened to identify additional studies.

A total of 158 publications were included. Because this was a narrative review, the evidence was synthesized qualitatively, without meta-analysis or formal risk-of-bias assessment.

## Figures and Tables

**Figure 1:**
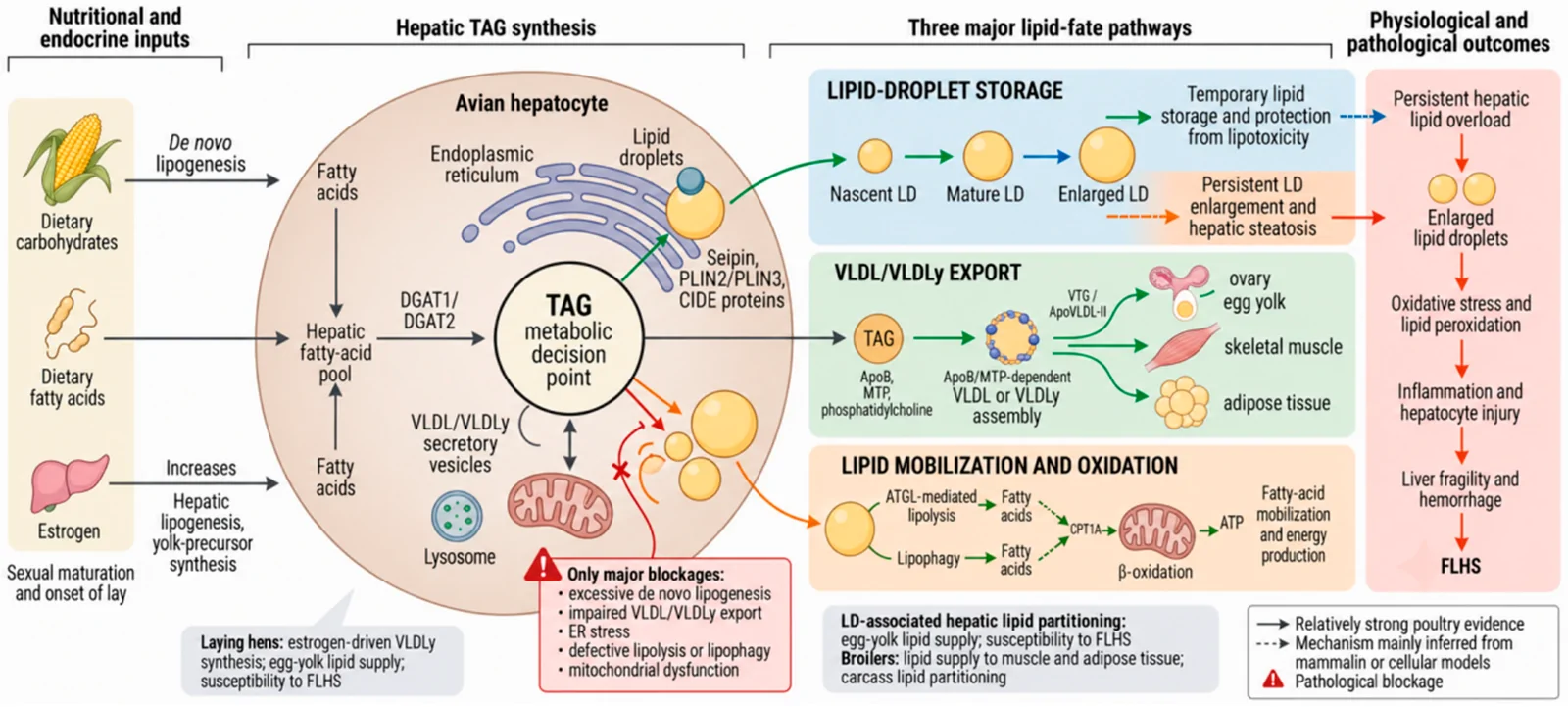
LD-associated lipid handling and liver-centered lipid flux in poultry. In poultry, the liver is the major site of de novo lipogenesis. Hepatic LDs participate in lipid storage, VLDL/VLDLy export, and lipid distribution to the ovary, skeletal muscle, and adipose tissue. Balanced LD-associated lipid handling may support egg production and appropriate intramuscular fat deposition, whereas excessive hepatic LD accumulation is a characteristic feature of fatty liver and metabolic dysfunction. This figure summarizes poultry lipid metabolism studies discussed in the main text and does not imply that all LD-related mechanisms have been causally established in poultry. The diagram separates the three principal fates of hepatic TAG: storage in LDs; ApoB/MTP-dependent VLDL/VLDLy export, including estrogen-driven VTG/ApoVLDL-II delivery to the ovary; and fatty-acid mobilization through ATGL-mediated lipolysis or lipophagy followed by CPT1A-dependent mitochondrial β-oxidation. Solid arrows denote processes with relatively strong poultry support, whereas dashed arrows denote mechanisms inferred mainly from mammalian or cellular models.

**Figure 2:**
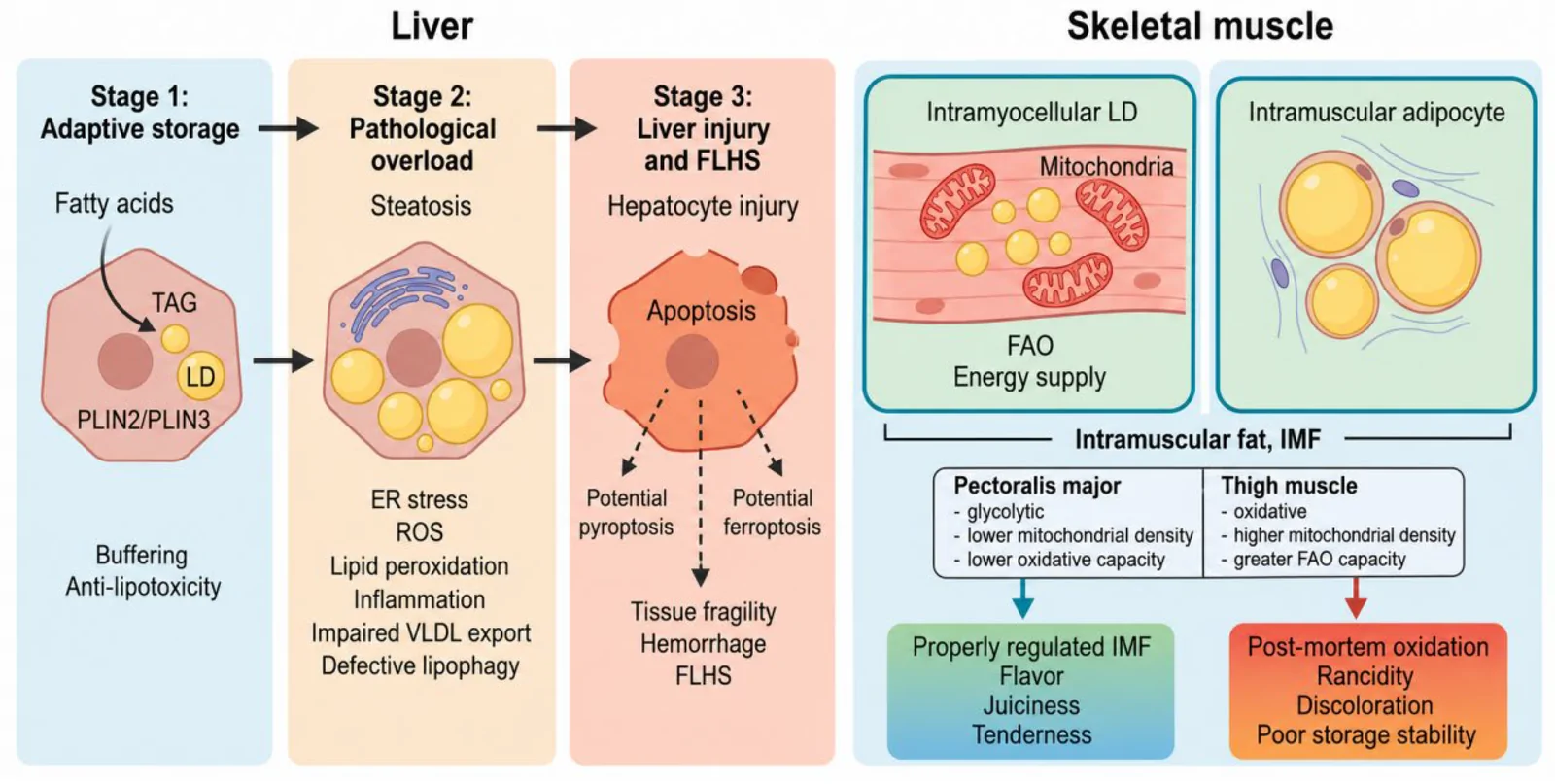
Divergent involvement of LDs in poultry fatty liver and muscle lipid deposition. Excessive hepatic LD accumulation is a hallmark of hepatic steatosis and fatty liver hemorrhagic syndrome (FLHS) and may be associated with enhanced de novo lipogenesis, TAG synthesis, PLIN2-associated LD expansion, impaired LD turnover, mitochondrial dysfunction, oxidative stress, ER stress, and inflammation. In skeletal muscle, properly regulated LD storage may contribute to desirable intramuscular fat deposition, whereas excessive or poorly coordinated lipid accumulation may impair muscle metabolism and meat quality. This figure integrates poultry studies with mechanistic concepts discussed in the main text. The muscle panel distinguishes intramyocellular LDs from adipocytes located in intramuscular connective tissue and contrasts the lower oxidative capacity of pectoralis major with the greater fatty-acid oxidative capacity of thigh muscle. Apoptosis is presented as the better-supported avian pathway, whereas pyroptosis and ferroptosis are shown as potential mechanisms requiring direct validation. Post-mortem lipid oxidation is linked to rancidity, discoloration, and poor storage stability.

**Figure 3:**
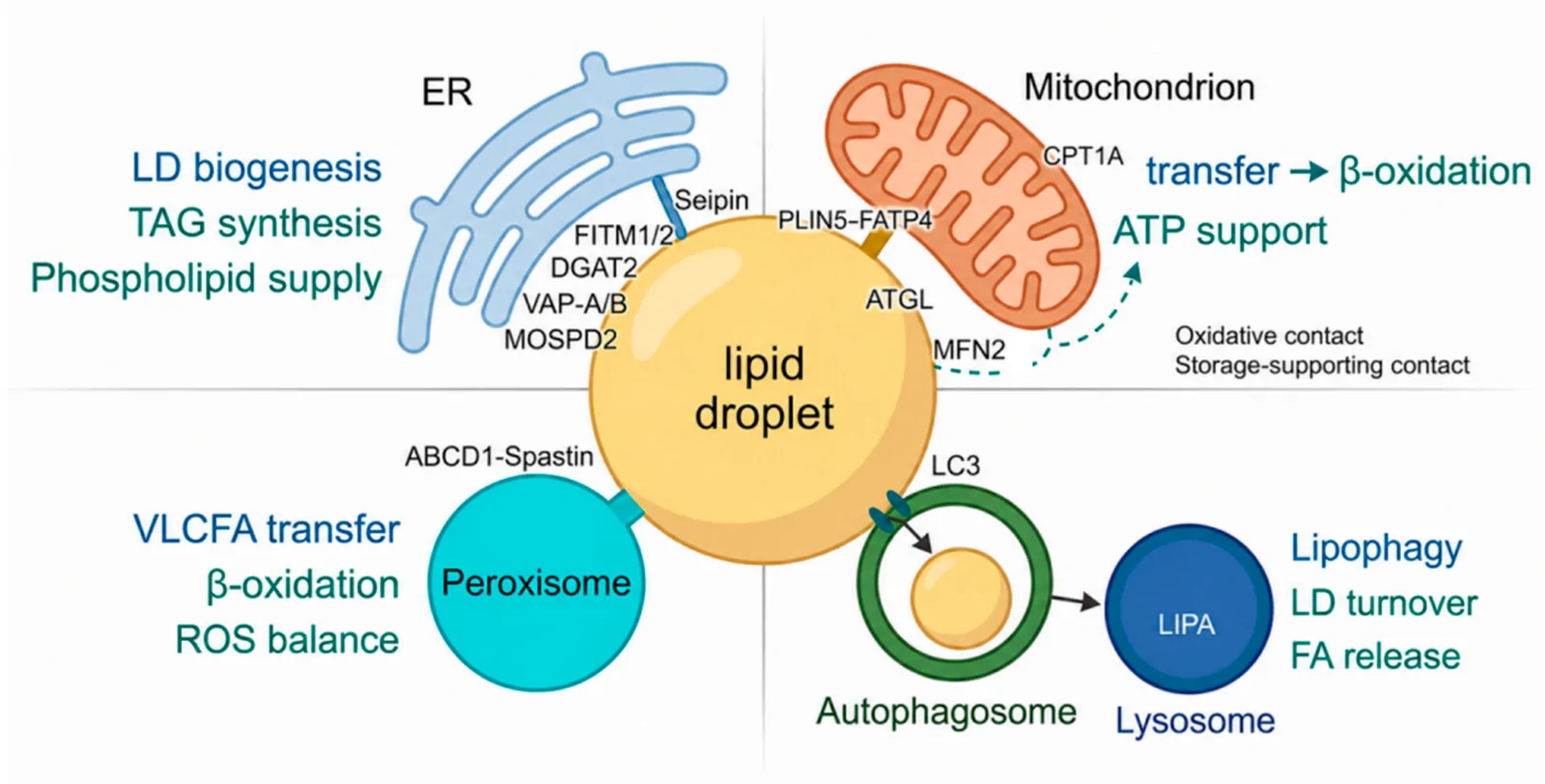
Potential LD–organelle interactions relevant to lipid fate in poultry cells. LDs can interact with multiple organelles to coordinate lipid synthesis, fatty-acid oxidation, redox balance, and lipid clearance. LD–ER contacts support LD biogenesis, TAG synthesis, and phospholipid supply. LD–mitochondria contacts may facilitate fatty-acid transfer to β-oxidation and support ATP-dependent lipid handling. LD–peroxisome contacts may support very-long-chain fatty-acid transfer, peroxisomal β-oxidation, and ROS balance. LD–lysosome interactions mediate lipophagy, LD turnover, and fatty-acid release. This figure integrates poultry lipid metabolism studies with mechanistic insights from mammalian and cellular models; poultry-specific validation of several contact-site mechanisms remains limited. Candidate contact-site components shown include seipin, FITM1/2, DGAT2, VAP-A/B, and MOSPD2 at ER–LD interfaces; PLIN5–FATP4 and candidate MFN2 at LD–mitochondria contacts, with ATGL on the LD surface and CPT1A on the mitochondrial outer membrane; ABCD1–spastin at LD–peroxisome contacts; and LC3/LIPA in lipophagy. The dashed MFN2 connection indicates a candidate mechanism requiring direct poultry validation.

**Figure 4:**
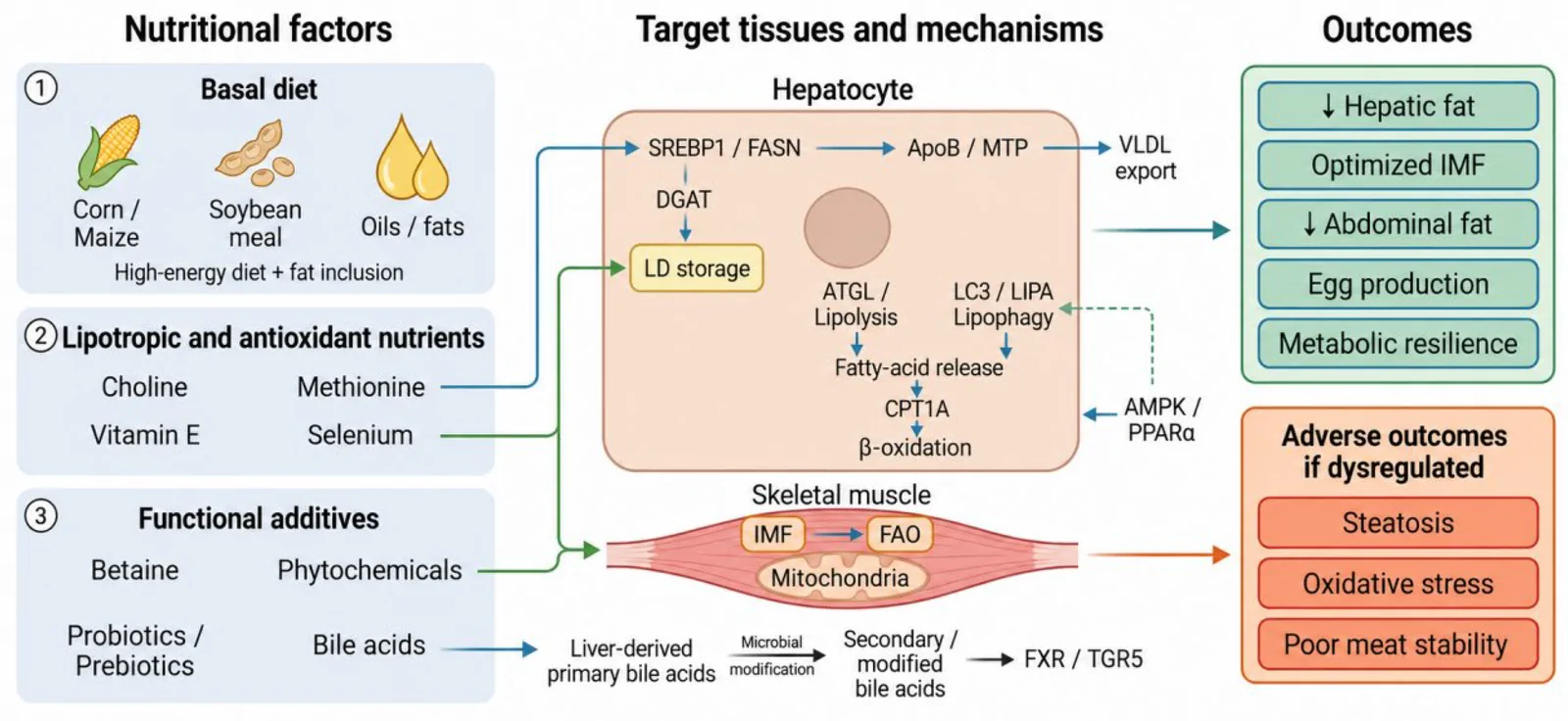
Nutritional strategies that may influence LD-associated lipid handling in poultry. Nutritional interventions, including balanced dietary energy, corn/maize-based energy supply, soybean meal-derived protein and amino acids, dietary fatty acid sources, choline, methionine, antioxidant nutrients, phytochemicals, probiotics, and bile acids, may regulate lipid metabolism in poultry liver and skeletal muscle. Betaine is considered here as a functional methyl donor studied in specific contexts rather than as a universal basal feed additive. These strategies may influence hepatic lipogenesis, ApoB/MTP-dependent VLDL export, ATGL-mediated lipolysis, LC3/LIPA-dependent lipophagy, AMPK/PPARα signaling, mitochondrial β-oxidation, and muscle lipid utilization; however, direct LD-specific evidence remains limited for many additives. Potential outcomes include reduced pathological hepatic and abdominal fat, optimized IMF, improved egg production, and greater metabolic resilience, whereas dysregulation may favor steatosis, oxidative stress, and poor meat stability. The bile-acid pathway distinguishes liver-derived primary bile acids from their microbial conversion to secondary or otherwise modified bile acids, and the dashed input to lipophagy denotes indirect evidence.

**Table 1:** Key Regulators of Lipid Droplets in Poultry.

Regulator/Class	Core Molecular Function	Relevance in Poultry Liver	Relevance in Skeletal Muscle	Current Evidence
Seipin/BSCL2	Organizes ER sites for neutral-lipid nucleation and promotes normal LD budding and maturation.	May determine the number and morphology of nascent hepatic LDs during high lipogenic flux.	Potentially controls myocellular LD biogenesis and prevents abnormal LD morphology.	Strong model-system evidence; direct poultry functional evidence is limited [34,37].
FITM1/2	Partitions neutral lipids within the ER and facilitates LD budding.	Candidate regulators of hepatic LD emergence and TAG storage.	FITM1 may be particularly relevant to muscle LD formation and energy storage.	Mainly mammalian and cell-model evidence [38].
DGAT1	Catalyzes TAG synthesis; supports re-esterification of preformed fatty acids and complete VLDL lipidation.	May favor VLDL/VLDLy particle maturation and limit cytosolic TAG retention when export capacity is adequate.	Contributes to TAG synthesis but its role in myocellular LD growth is incompletely defined.	Mechanistic evidence is mainly mammalian; avian isoform-specific function remains unresolved [46,47].
DGAT2	Uses nascent diacylglycerol and de novo synthesized fatty acids; supports local TAG synthesis at ER-LD interfaces.	Likely contributes strongly to hepatic TAG storage and LD expansion during active lipogenesis.	May promote local TAG synthesis and myocellular LD growth.	Mechanistic evidence is mainly mammalian; avian isoform-specific validation remains limited [33,46,47].
PLIN2	Stabilizes mature LDs and regulates lipase and autophagy access to the LD surface.	May favor hepatic LD persistence and lipid retention under sustained lipogenic pressure.	May stabilize intramyocellular LDs, especially when oxidative utilization is insufficient.	Predominantly mammalian mechanistic evidence; poultry-specific functional validation remains limited [43,45,48].
PLIN3	Rapidly coats nascent/small LDs and participates in early LD maturation and protein targeting.	Potential regulator of newly formed hepatic LDs during rapid TAG synthesis.	May regulate small, dynamic LDs within myofibers.	Predominantly mammalian and structural evidence; poultry function is largely unknown [44].
PLIN5	Coordinates LD storage, lipase access, and coupling with mitochondria in oxidative cells.	Candidate regulator of controlled fatty-acid release and hepatic oxidative adaptation.	Potentially links intramyocellular LDs to mitochondrial fatty-acid utilization.	Strong mammalian evidence; direct poultry contact-site evidence remains scarce [49,50,51,52,53].
CIDEA/CIDEC	Promotes LD growth, lipid transfer, and LD fusion.	May contribute to enlargement of hepatic LDs during lipid overload.	May influence adipogenic lipid storage in intramuscular connective tissue.	Poultry transcriptomic associations exist; direct LD-level validation is limited [39,40,54].
ATGL/PNPLA2-ABHD5	Initiates TAG hydrolysis and releases fatty acids from LDs.	Controls the balance between hepatic lipid retention, oxidation, and export.	Supplies fatty acids from intramyocellular LDs for oxidation.	Functional poultry evidence remains less developed than mammalian evidence [55].
HSL/LIPE	Hydrolyzes diacylglycerol and cooperates with ATGL during lipid mobilization.	May contribute to hepatic LD turnover under energy demand.	Supports lipid mobilization in muscle-associated adipocytes and potentially myofibers.	Predominantly mammalian mechanistic evidence; poultry-specific validation remains limited [55].
LC3-LIPA lipophagy pathway	Delivers LDs to lysosomes and hydrolyzes neutral lipids through lysosomal acid lipase.	Potential defense against chronic hepatic LD overload and FLHS.	Its contribution to myocellular LD turnover is largely unknown.	Emerging avian evidence; direct flux measurements are uncommon [56,57,58].
ApoB-MTP pathway	Assembles and lipidates VLDL/VLDLy as an alternative fate for hepatic TAG.	Critical for hepatic lipid export to the ovary and peripheral tissues; failure favors LD retention.	Influences muscle lipid supply indirectly through circulating lipoproteins.	Established avian physiological relevance, but its direct coordination with LD dynamics requires further study [1,3].

## Data Availability

No new data were created or analyzed in this study. Data sharing is not applicable.
